# Pre-Resection Endoscopic Features in T1 Colorectal Cancer Selected for Salvage Surgery After ESD/EID: An Exploratory Comparison of SM1 and SM2–3 Invasion

**DOI:** 10.3390/jcm15197634

**Published:** 2026-10-02

**Authors:** Kasper Maryńczak, Julia Adamkiewicz, Jacek Kabziński, Aleksandra Osielczak, Przemysław Kasprzyk, Adam Dziki, Michał Spychalski, Ireneusz Majsterek, Łukasz Dziki

**Affiliations:** 1 Department of Clinical Chemistry and Biochemistry, Medical University of Lodz, 92-213 Lodz, Poland; kasper.marynczak@umed.lodz.pl (K.M.); jacek.kabzinski@umed.lodz.pl (J.K.); ireneusz.majsterek@umed.lodz.pl (I.M.); 2 Department of General and Oncological Surgery, Medical University of Lodz, 92-213 Lodz, Poland; aleksandra.osielczak@student.umed.lodz.pl (A.O.); adam.dziki@umed.lodz.pl (A.D.); michal.spychalski@umed.lodz.pl (M.S.); lukasz.dziki@umed.lodz.pl (Ł.D.); 3 Brzeziny Specialist Hospital, 95-060 Brzeziny, Poland; kasprzykprzemek@gmail.com; 4 Military Medical Academy Memorial Teaching Hospital of the Medical University of Lodz—Central Veteran Hospital, 90-549 Lodz, Poland

**Keywords:** colorectal cancer, endoscopic submucosal dissection, surgical guidelines, risk stratification, submucosal invasion, Paris classification, pit pattern

## Abstract

**Background/Objectives:** Accurate assessment of submucosal invasion is important when selecting patients with early colorectal cancer for organ-preserving treatment. Endoscopic submucosal dissection (ESD) and endoscopic intermuscular dissection (EID) enable en bloc resection and detailed histopathological assessment. This study explored whether routinely recorded pre-resection endoscopic characteristics differed between SM1 and SM2–3 lesions within a selected cohort of patients who underwent salvage surgery after non-curative endoscopic resection. **Methods:** We retrospectively analyzed 43 patients with T1 colorectal cancer who underwent ESD or EID and subsequently underwent salvage surgery because of non-curative histopathological findings. Among the 43 included patients, 37 underwent ESD and 6 underwent EID. Pre-resection Paris morphology and pit-pattern classification were compared according to final histopathological depth of submucosal invasion. Exploratory univariable logistic regression was used to examine associations with SM2–3 invasion. **Results:** Five patients had SM1 invasion and 38 had SM2–3 invasion. No statistically significant differences in the evaluated baseline or endoscopic variables were observed between the two groups. Exploratory regression estimates were imprecise, with wide confidence intervals, reflecting the small number of SM1 cases. **Conclusions:** In this selected cohort of patients undergoing salvage surgery after non-curative ESD/EID, Paris morphology and pit-pattern classification did not show statistically significant differences between SM1 and SM2–3 lesions. Because curatively treated patients were not included and only five patients had SM1 invasion, these findings do not estimate the diagnostic performance of these classifications in the broader pre-resection population and should be considered hypothesis-generating.

## 1. Introduction

Colorectal cancer (CRC) remains a major global health burden and is among the most frequently diagnosed malignancies and leading causes of cancer-related death worldwide. Recent global estimates confirm persistently high incidence and mortality across regions, despite advances in screening and treatment strategies [1]. Projections indicate that the global age-standardized prevalence rate of CRC among the total population will reach 145.82 per 100,000 individuals by 2040, which constitutes an 8.15% increase compared to the rates documented in 2021 [2]. Based on the 2022 GLOBOCAN estimates described online at the Global Cancer Observatory (https://gco.iarc.who.int; accessed on 14 April 2026) CRC was predicted to be the third most common type of cancer globally, with over 1.9 million new cases and more than 900,000 deaths annually, making it the second highest mortality cancer worldwide [3,4]. Furthermore, it is important to highlight the rise in early-onset CRC, with increasing incidence among adults younger than 55 years, which has intensified efforts to optimize early detection and risk stratification [5]. Rectal cancer constitutes a clinically distinct subgroup of CRC because of its pelvic anatomy, implications for multimodal therapy, and the importance of organ preservation and functional outcomes [6]. Diagnosis is established by clinical interview, Fecal Occult Blood Test endoscopic biopsy and histological confirmation. Staging includes cross-sectional imaging of the chest, abdomen and pelvis for distant disease and dedicated pelvic MRI for locoregional nodal metastases assessment. Serum carcinoembryonic antigen (CEA) is measured at baseline primarily for prognostic assessment and as a reference for post-treatment surveillance; it is not used to establish the diagnosis and does not reliably predict response to neoadjuvant therapy [7].

Treatment pathways for rectal cancer depend on stage and risk profile. Locally advanced disease is managed using multimodal regimens that combine systemic therapy, radiotherapy, and total mesorectal excision, with increasing use of total neoadjuvant therapy and response-adapted strategies in selected patients [6,8]. For early rectal neoplasia and carefully selected T1 cancers without clinical evidence of nodal or distant metastasis, local excision techniques aim to provide oncologic safety while minimizing morbidity. These approaches include transanal platforms such as transanal endoscopic microsurgery and transanal minimally invasive surgery, as well as endoscopic resection techniques [9,10]. Within endoscopy, endoscopic mucosal resection (EMR) and endoscopic submucosal dissection (ESD) represent the principal local resection methods for superficial lesions and early cancers [11,12].

ESD has gained prominence because it enables en bloc resection irrespective of lesion size, which improves histopathologic assessment of lateral and vertical margins and reduces local recurrence compared with piecemeal EMR in appropriate settings [11,12,13]. However, colorectal ESD is technically complex, requires considerable expertise, and is associated with longer procedure times and a higher risk of complications, particularly perforation. These challenges have limited its widespread use outside in specialist hospitals that treat many such cases and have highly experienced endoscopists [11,12,13]. Because the oncologic value of ESD in T1 rectal cancer depends on achieving curative (low-risk) histology, accurate pre-resection characterization is essential. In routine practice, a substantial proportion of lesions resected by ESD ultimately demonstrate high-risk features, leading to recommendations for additional surgery despite technically successful endoscopic resection [14,15]. This creates a clinically relevant dilemma: ESD may function as a definitive organ-preserving treatment in a minority of cases but as a staging or selection procedure in many others.

Current selection for ESD is primarily based on endoscopic morphology and optical diagnosis. Guideline recommendations emphasize lesion size, laterally spreading tumor morphology, macroscopic appearance (Paris classification), and assessment of surface and vascular patterns using high-definition white light imaging and dye-based or virtual chromoendoscopy [13,16]. Classification systems including pit pattern analysis and narrow-band imaging-based schemes (e.g., JNET) can achieve good diagnostic performance for identifying lesions with overt deep submucosal invasion, supporting referral to surgery rather than endoscopic cure attempts [17,18]. However, the clinically decisive distinction in T1 cancer management is often not “no invasion versus deep invasion,” but superficial submucosal invasion (SM1) versus deeper invasion (SM2–3). This distinction is central because deep invasion is strongly associated with lymph node metastasis risk and typically reclassifies a lesion as high-risk after endoscopic resection [15,19]. The precision of optical diagnosis for predicting invasion depth along this SM1 to SM2–3 continuum remains limited, and pre-ESD assessment may not reliably anticipate the final histopathologic risk profile [13,15,19]. Consequently, patients who appear eligible for curative endoscopic therapy based on contemporary endoscopic criteria may still require completion surgery because high-risk features are detected only after the specimen is examined.

Given these limitations, there is growing interest in complementary biomarkers that could improve pre-treatment stratification beyond morphology alone. At present, no blood-based biomarker is validated or recommended in clinical guidelines to select T1 rectal cancer patients for ESD. Nevertheless, liquid biopsy approaches, including circulating tumor DNA (ctDNA), cell-free DNA methylation signatures, circulating microRNAs, and other analytes, have shown promise for CRC detection and risk assessment in broader contexts [20,21,22,23]. Notably, some blood-based CRC screening assays have received regulatory approval in specific jurisdictions, illustrating rapid progress in assay development and implementation pathways. However, such tests still produce high rates of false positives [23]. Therefore, translating blood-based biomarkers from population screening or metastatic monitoring into a tool that can discriminate superficial from deep submucosal invasion in early rectal cancer is scientifically and clinically challenging, in part because early lesions may shed limited tumor-derived material into the circulation [20,21,22]. High-quality prospective studies that link biomarker signatures with precise histopathologic invasion depth and established high-risk features are needed before such tests can inform ESD candidacy.

Against this background, the present study aimed to explore whether routinely recorded pre-resection endoscopic characteristics differed between SM1 and SM2–3 lesions within a selected cohort of patients who underwent salvage surgery following non-curative ESD/EID. The study was not designed to validate the diagnostic accuracy of these classifications in an unselected population of patients considered for primary endoscopic treatment.

## 2. Materials and Methods

### 2.1. Patients and Endoscopic Procedure

A retrospective analysis was conducted on a database of 43 patients diagnosed with early colorectal cancer who underwent Endoscopic Submucosal Dissection (ESD) or Endoscopic Intermuscular Dissection (EID) and were subsequently qualified for salvage surgical treatment due to suspected non-curative endoscopic resection. The study period was 4 years. All 43 patients were diagnosed with T1 colorectal cancer, 6 (13.95%) underwent EID and 37 (86.05%) underwent ESD. All endoscopic procedures were performed at a single referral center by an experienced endoscopist performing over 300 ESD procedures annually. The procedures were conducted using a high-definition video endoscopy system from Pentax Medical (Tokyo, Japan), utilizing EC34-i10c series videocolonoscopes equipped with HD+ CMOS sensors (Pentax Medical, Tokyo, Japan). During the procedure, each lesion was assessed in detail regarding location and size (mm); for rectal lesions, the distance from the anal verge was measured. The morphological characteristics of the lesion were defined according to the Paris classification and LST type (Granular/Non-Granular), while the surface and vascular structure (pit pattern) were evaluated using virtual chromoendoscopy i-SCAN (SE and TE modes) and Optical Enhancement (OE). En bloc resection was performed using needle-type knives and an endoscopic cap. Immediately after the procedure, the obtained tissue specimen was pinned onto a corkboard to prevent retraction, then fixed in a 4% formalin solution and submitted for histopathological evaluation.

### 2.2. Surgical Treatment and Histopathological Analysis

The indication for surgery was the presence of at least one histopathological risk factor for lymph node metastasis or local recurrence, defined as: positive vertical or horizontal resection margins (R1/Rx); deep submucosal invasion (SM2 or SM3 according to the Kikuchi classification, or Haggitt level 4); presence of lymphovascular invasion (LVI); high-grade tumor budding (Bd 2/3); or poor histological differentiation (G3) within the cancer focus. All patients in the analyzed group underwent secondary surgical treatment at the same center. Colorectal resection procedures were performed using either open or laparoscopic approaches, depending on clinical indications and lesion location. Postoperative specimens were also preserved and stored in a 4% formalin solution. The final histopathological analysis, including both the ESD/EID specimen and the surgical material, involved the assessment of submucosal invasion depth, resection margin status (horizontal and vertical), histological grade, lymphovascular invasion (LVI), tumor budding, and the potential presence of metastases in retrieved lymph nodes. The aim of the present analysis was to explore whether routinely recorded pre-resection clinical and endoscopic characteristics differed between lesions with superficial (SM1) and deeper (SM2–3) submucosal invasion within this selected cohort of patients who underwent salvage surgery following non-curative ESD/EID.

### 2.3. Statistical Analysis

All analyses were conducted using R (version 4.5.1; R Foundation for Statistical Computing, Vienna, Austria).

Continuous variables were assessed for distribution and are presented as medians with interquartile ranges (IQR), given the small sample size and non-normal distribution. Categorical variables are presented as counts and percentages.

Comparisons between patients with shallow submucosal invasion (SM1) and deep submucosal invasion (SM2–3) were performed using the Wilcoxon rank-sum test for continuous variables and Fisher’s exact test for categorical variables.

To explore associations between pre-resection endoscopic characteristics and deep submucosal invasion (SM2-–3), univariable logistic regression models were fitted. The outcome variable was SM2–3 invasion (yes/no). Predictor variables included tumor size (analyzed per 10 mm increase), rectal location (rectum vs. colon), Paris morphology (categorized), and pit pattern V (yes/no). Odds ratios (ORs) with 95% confidence intervals (CIs) were calculated.

Given the marked imbalance between SM1 and SM2–3 groups and the presence of only five SM1 cases, logistic regression analyses were considered exploratory. No multivariable model was fitted. Odds ratios and 95% confidence intervals were used to describe the direction and uncertainty of associations rather than to establish predictive performance. Sensitivity, specificity, predictive values, and area under the receiver operating characteristic curve were not calculated because the selected cohort was not suitable for formal diagnostic accuracy assessment.

All statistical tests were two-sided. A *p*-value < 0.05 was considered statistically significant.

### 2.4. AI Statement

Generative artificial intelligence (ChatGPT, OpenAI) was used during manuscript preparation to support English-language editing, improve clarity and organization of the text, and assist in drafting and restructuring selected sections of the manuscript. The tool was also used to assist in identifying potentially relevant literature for consideration by the authors. Generative AI was not used for study design, patient selection, data collection, generation of study data, statistical analysis, or generation of the reported results. All AI-assisted content, interpretations, and references were critically reviewed, verified, and revised by the authors, who take full responsibility for the accuracy and integrity of the final manuscript.

## 3. Results

The study cohort comprised 43 patients with T1 colorectal cancer who underwent endoscopic resection, including 37 patients treated with ESD and 6 treated with EID. On final histopathology, SM1 invasion was present in 5 patients (11.6%), whereas SM2–3 invasion was present in 38 patients (88.4%). Baseline clinical and endoscopic characteristics stratified according to the depth of submucosal invasion are presented in Table 1. Median age was 60 years (IQR 50–65) in the SM1 group and 65 years (IQR 61–67) in the SM2–3 group (*p* = 0.262). The proportion of male patients was similar between groups (4/5 [80.0%] vs. 27/38 [71.1%], *p* = 1.000). Median body mass index was 25.39 kg/m^2^ (IQR 21.80–26.54) in SM1 and 25.85 kg/m^2^ (IQR 23.73–29.14) in SM2–3 (*p* = 0.776). Rectal location was observed in 2/5 (40.0%) patients with SM1 and 21/38 (55.3%) patients with SM2–3 (*p* = 0.650). Median lesion size was 40 mm (IQR 25–45) in SM1 and 30 mm (IQR 20.5–48.8) in SM2–3 (*p* = 0.690). No statistically significant between-group differences were observed in the baseline clinical or endoscopic variables evaluated.

Exploratory univariable logistic regression was performed to examine associations between selected pre-resection characteristics and SM2–3 invasion (Table 2). None of the evaluated associations reached statistical significance. Tumor size (per 10 mm) yielded an OR of 1.06 (95% CI 0.75–1.78; *p* = 0.773), rectal location an OR of 1.85 (95% CI 0.28–15.31; *p* = 0.525), Paris flat morphology an OR of 2.57 (95% CI 0.38–21.38; *p* = 0.332), Paris depressed morphology an OR of 0.22 (95% CI 0.02–5.39; *p* = 0.259), and pit pattern V an OR of 2.33 (95% CI 0.27–16.62; *p* = 0.395). Confidence intervals were wide, indicating substantial uncertainty in the estimates. Given the small number of SM1 cases, these analyses should be interpreted as exploratory and not as evidence of absence of association or as formal estimates of diagnostic performance.

**Figure 1:**
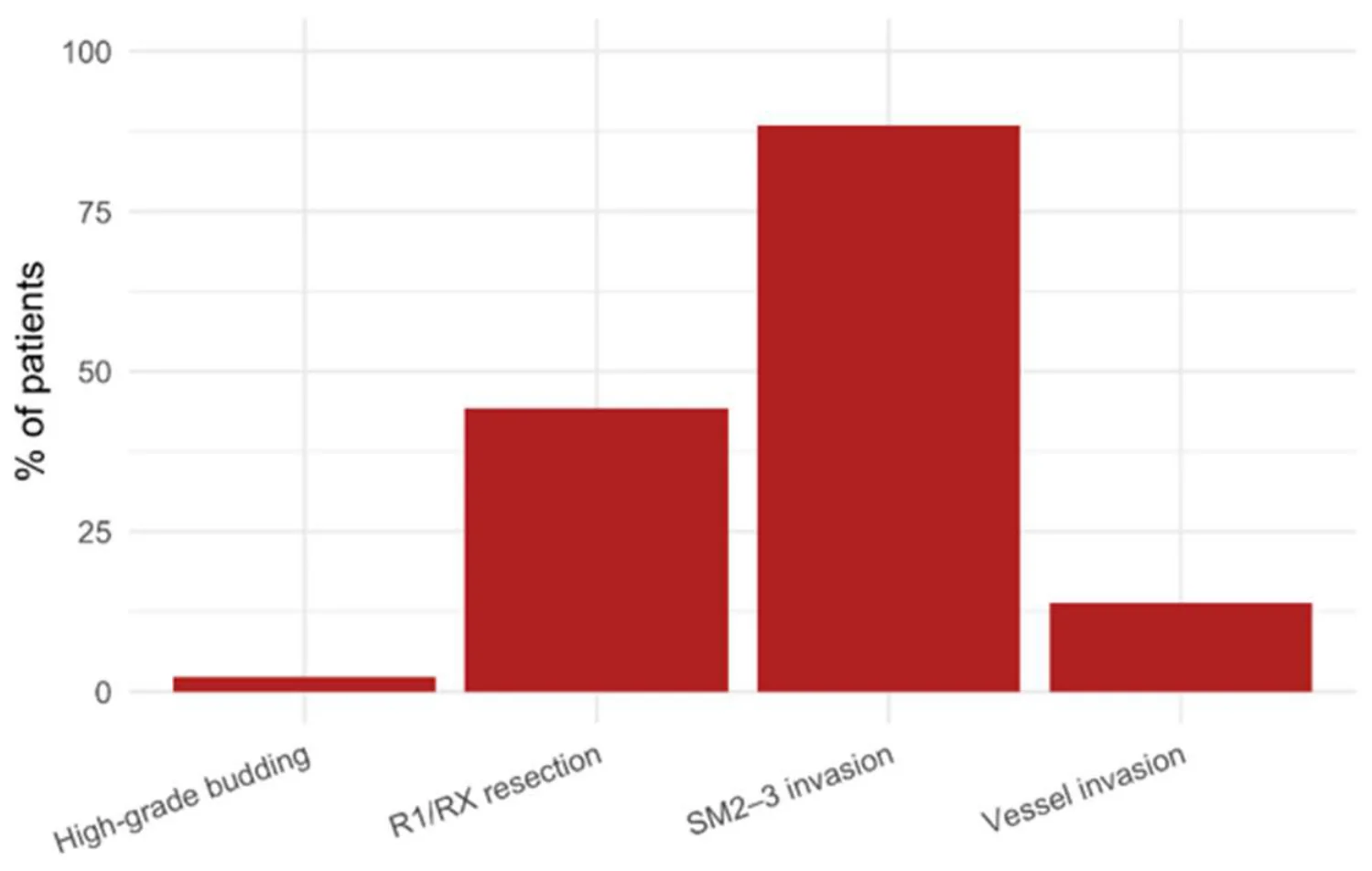
High-risk histopathological features after ESD/EID in T1 colorectal cancer. Bars represent the proportion of patients with high-grade tumor budding, R1/Rx resection status, deep submucosal invasion (SM2–3), and lymphovascular invasion. ESD—endoscopic submucosal dissection.

Histopathological adverse features were frequent in this selected cohort. Deep submucosal invasion (SM2–3) was observed in 38/43 patients (88.4%), R1/Rx resection status in 19/43 (44.2%), lymphovascular invasion in 6/43 (14.0%), and high-grade tumor budding in 1/43 (2.3%) (Figure 1). All 43 patients underwent salvage surgical treatment according to the study inclusion pathway.

**Figure 2:**
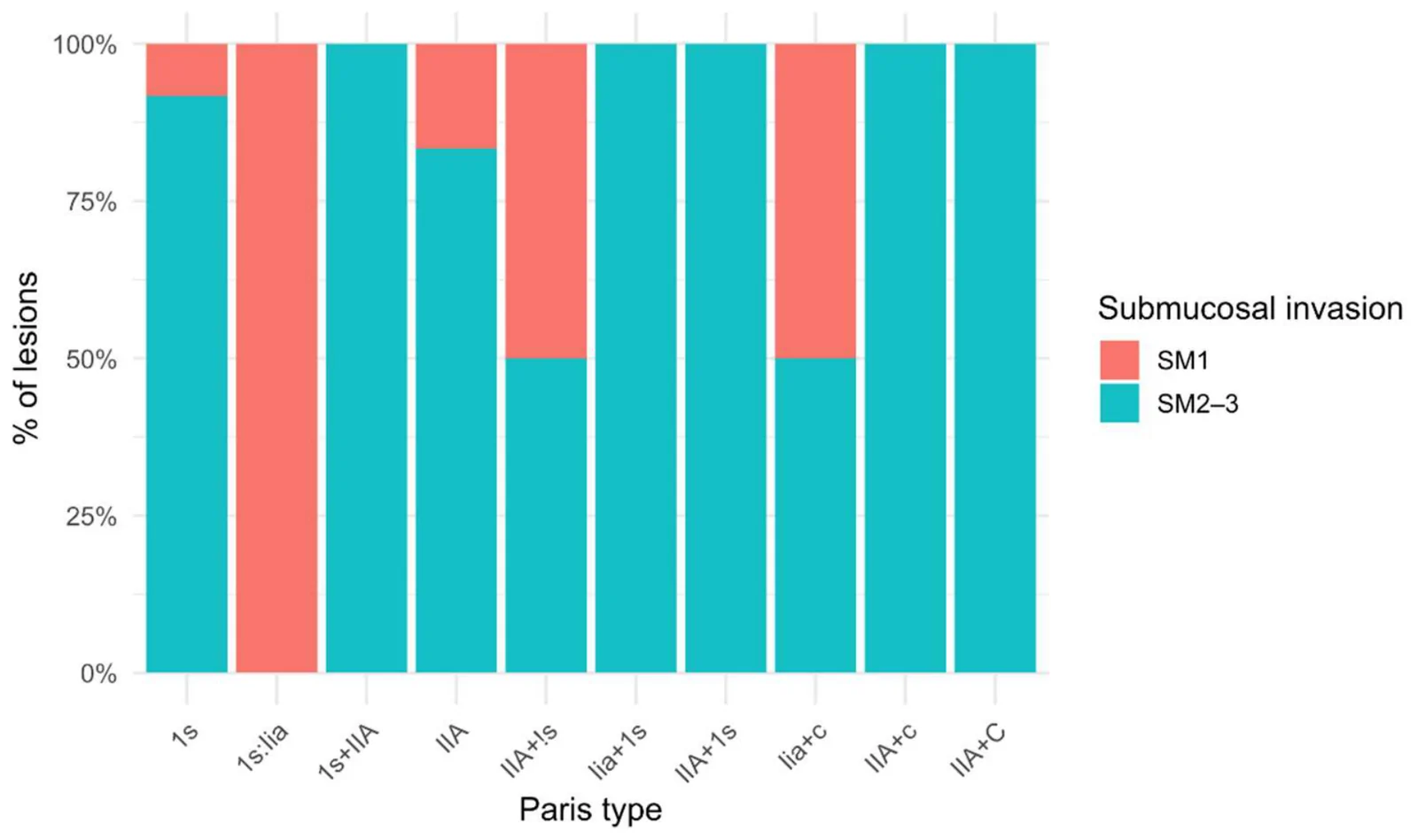
Distribution of depth of submucosal invasion according to Paris classification. Stacked bars show the percentage of lesions with SM1 versus SM2–3 invasion across Paris morphologies, demonstrating substantial overlap between groups.

**Figure 3:**
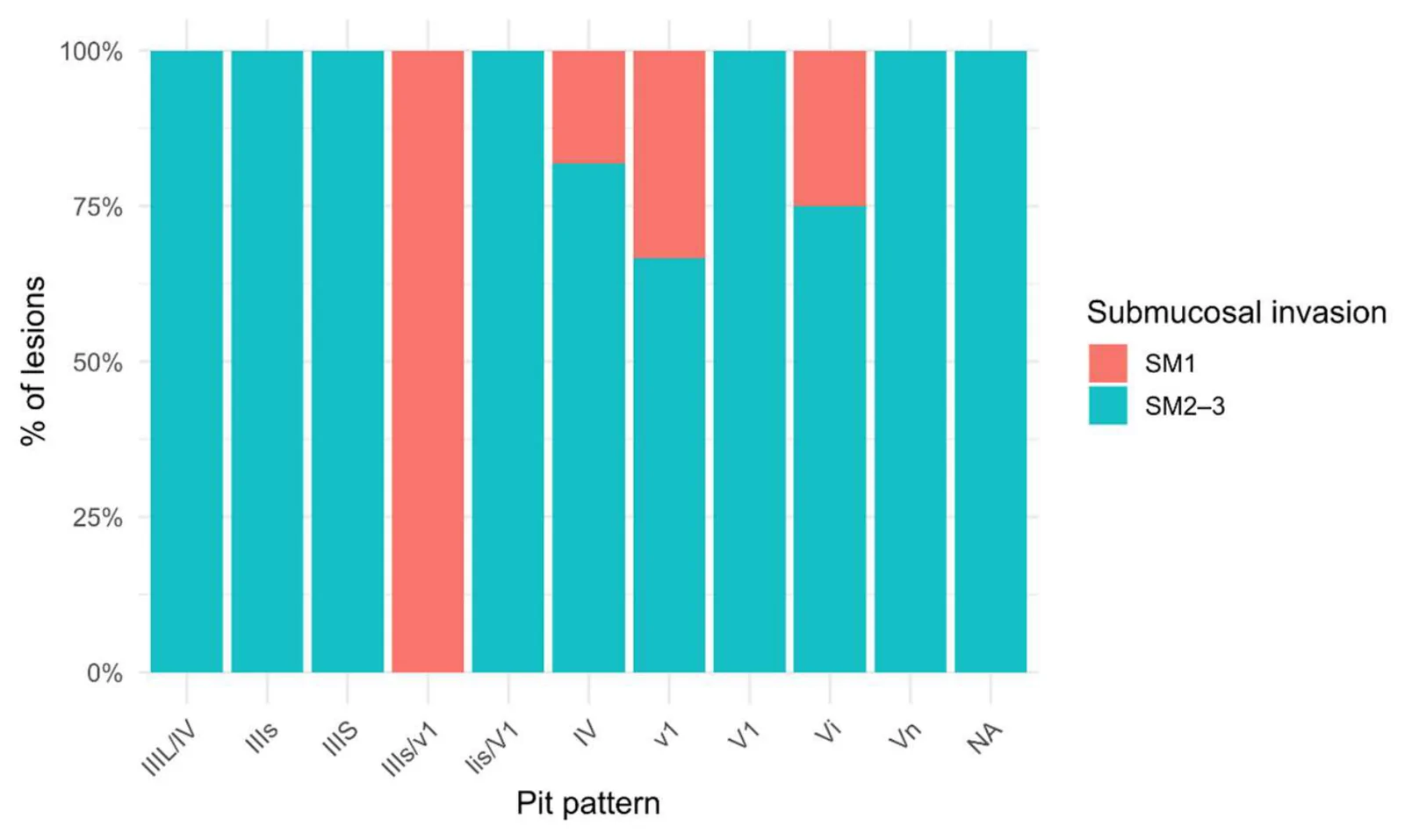
Distribution of depth of submucosal invasion according to pit pattern. Stacked bars show the percentage of lesions with SM1 versus SM2–3 invasion across pit pattern categories, without a category that reliably identifies SM2–3 invasion.

The distributional plots in this cohort did not indicate clear discriminatory performance of macroscopic and microstructural endoscopic classifications. Paris types showed substantial overlap between SM1 and SM2–3 lesions (Figure 2), including lesions with a flat appearance that were nevertheless observed among cases with deeper invasion. Likewise, pit pattern categories were present in both SM1 and SM2–3 groups, without a consistent predominance of any specific pattern in SM2–3 lesions (Figure 3). Taken together, these observations indicate substantial overlap in pre-resection Paris morphology and pit-pattern categories between SM1 and SM2–3 lesions within this markedly imbalanced cohort.

## 4. Discussion

The present study should be interpreted as an exploratory analysis of a selected subgroup of patients with T1 colorectal cancer rather than as a validation study of pre-resection endoscopic classification systems. All included patients underwent ESD/EID and subsequently underwent surgical treatment after the endoscopic resection was classified as non-curative on histopathological grounds. Consequently, the study population was enriched for adverse pathological features and excluded patients with curative endoscopic resection. The predominance of SM2–3 invasion observed in this cohort should therefore be interpreted in the context of this selection pathway and should not be extrapolated to the overall population of patients with T1 colorectal cancer undergoing pre-resection endoscopic assessment.

Within this selected cohort, no statistically significant differences in the evaluated clinical or endoscopic characteristics were observed between patients with SM1 and SM2–3 invasion. Exploratory univariable regression analyses similarly did not demonstrate statistically significant associations between lesion size, location, Paris morphology, or pit pattern and SM2–3 invasion. However, only five patients had SM1 invasion, and the corresponding effect estimates were imprecise, as reflected by the wide confidence intervals. These findings therefore indicate substantial uncertainty within the present dataset rather than evidence that the evaluated endoscopic features have no predictive value.

This distinction is important because established optical and morphological classification systems have demonstrated clinically relevant diagnostic performance in larger studies and form part of current guideline-based assessment of colorectal lesions [13,16,17,18,19]. Our findings should therefore not be interpreted as challenging the validity of these classifications or current recommendations for pre-resection lesion assessment. Instead, they describe a narrower clinical situation: lesions that underwent endoscopic resection but subsequently proved non-curative on histopathological evaluation. Within this selected subgroup, substantial overlap in pre-resection morphology was observed between lesions with superficial and deeper submucosal invasion.

Because patients with curative endoscopic resection were not included, the present study cannot estimate the sensitivity, specificity, positive or negative predictive values, or overall diagnostic accuracy of Paris morphology or pit-pattern classification in the broader population of patients considered for ESD/EID. The findings are therefore relevant primarily to describing residual uncertainty within a selected group of patients who ultimately entered a salvage-surgery pathway and cannot be used to determine the overall performance of these classifications at the initial point of treatment selection.

The marked imbalance between the study groups represents an additional methodological limitation. With only five SM1 cases compared with 38 SM2–3 cases, statistical power was limited and logistic regression estimates were inherently unstable. The wide confidence intervals should therefore be interpreted as reflecting uncertainty in the estimated associations rather than as evidence of absence of an association. For this reason, the regression analyses were considered exploratory, and no multivariable modelling was attempted. Larger prospective studies including consecutive patients with T1 colorectal cancer and a broader distribution of invasion depths are required to estimate more reliably the association between individual endoscopic features and histopathological invasion depth.

Although the present cohort cannot be used to estimate how frequently endoscopically treated T1 colorectal cancers require subsequent surgery, it illustrates the importance of definitive histopathological assessment after en bloc resection in patients with non-curative findings. Histological characteristics such as lymphovascular invasion, poor differentiation, tumor budding, resection-margin involvement, and depth of submucosal invasion contribute to decisions regarding additional treatment [15,17,19]. Previous studies have shown that completion surgery after non-curative ESD is feasible and can be performed without necessarily compromising short-term surgical outcomes [24,25]. In the present cohort, all patients proceeded to surgical treatment by definition of the study population. Therefore, the observed frequency of adverse histopathological features should not be interpreted as the expected rate of surgical referral after ESD/EID in an unselected population. Rather, these findings illustrate the role of endoscopic resection in providing definitive histopathological information in patients whose lesions subsequently prove to meet criteria for additional treatment.

Importantly, the potential clinical value of pre-resection endoscopic stratification in this setting extends beyond its ability to predict final histopathological invasion depth. Even when definitive decisions regarding salvage surgery are ultimately based on post-resection pathology, preoperative endoscopic classification may still inform the treatment pathway. Lesions with features raising concern for deeper invasion may prompt more explicit pre-procedural counseling regarding the possibility that endoscopic therapy may not be definitive and that subsequent surgical treatment could be required. Such findings may also support earlier multidisciplinary discussion, particularly in patients for whom the balance between organ preservation and oncological risk is clinically complex. In addition, standardized pre-resection endoscopic descriptors provide structured variables that could be incorporated into future multidimensional risk models rather than being interpreted in isolation [13,17,18,19,26].

A key limitation of current preoperative stratification is that the decisive predictors of LNM risk; lymphovascular invasion; poor differentiation; high-grade tumor budding; and deep submucosal invasion remain histopathologic constructs that are typically unavailable until after en bloc resection [17,19]. Tumor budding, in particular, has been repeatedly linked to nodal risk in submucosally invasive colorectal cancer, reinforcing why “low-risk” classification cannot rely on surface morphology alone [27]. This creates an intrinsic diagnostic bottleneck: endoscopy determines resectability and provides probabilistic suspicion of invasion depth, while pathology provides the definitive risk stratification that drives the decision for completion surgery. Accordingly, in patients in whom adverse histopathological features are identified after endoscopic resection, management may follow a two-step pathway, with endoscopic resection providing both local treatment and definitive pathological staging, followed by surgery when indicated.

From this perspective, the residual uncertainty observed in the present cohort may be clinically relevant not because it invalidates current endoscopic classifications, but because it identifies an area in which additional information could improve pre-treatment risk estimation. Even in patients who ultimately prove to have pathological high-risk features, pre-resection endoscopic stratification may support more informed doctor–patient communication regarding the possibility of non-curative endoscopic treatment and subsequent salvage surgery. It may also help identify cases that warrant early multidisciplinary review and provide structured clinical variables for future multidimensional risk-prediction models. Such models could integrate conventional morphology and optical classification with patient characteristics, lesion-related variables, artificial intelligence-assisted image analysis, and molecular biomarkers [20,21,22,28,29,30,31,32]. In the future, this type of integrated approach could potentially estimate the probability of non-curative endoscopic resection or subsequent salvage surgery before treatment, thereby supporting shared decision-making and treatment planning. However, this concept remains hypothetical and requires validation in prospective cohorts including both curatively and non-curatively treated patients.

Among the approaches that could contribute to such multidimensional pre-resection risk assessment, artificial intelligence (AI) systems trained on endoscopic images have shown promise for predicting the depth of submucosal invasion and distinguishing shallow from deep invasion patterns [28]. Although these studies are encouraging, they require rigorous external validation, prospective testing, and careful assessment of generalizability across endoscopy platforms, imaging modalities, and operator technique. In parallel, standardized optical assessment and structured reporting, as emphasized in technical and practice updates, may reduce variability and improve the reproducibility of preoperative impressions [26].

Blood-based biomarkers and liquid biopsy strategies represent another potential adjunct to endoscopic evaluation, but their clinical readiness for ESD selection in T1 rectal cancer remains limited. Systematic reviews describe the growing landscape of circulating tumor DNA, methylation assays, and other analytes for colorectal cancer detection and monitoring [20,21,22]. However, most biomarker development has focused on screening, prognosis, and minimal residual disease rather than the specific pre-ESD question of SM1 versus SM2–3 invasion. cfDNA methylation panels can discriminate cancer from benign disease with promising accuracy in some settings [29], and methylation profiles are also being explored for treatment monitoring [30]. Additional studies continue to report diagnostic and prognostic performance of methylation signatures in colorectal cancer [31]. MicroRNA-based signatures may offer complementary information, but their translation is hampered by assay variability and the need for robust validation [32]. A major biological constraint is that very early invasive tumors may shed limited circulating material, which could reduce sensitivity precisely in the population where endoscopic triage is most needed. Therefore, at present, blood biomarkers should be viewed as investigational adjuncts rather than tools that can replace endoscopic staging for ESD candidate selection.

This study has several important limitations. First, it was retrospective and conducted at a single high-volume referral center, which limits external validity. More importantly, the cohort was selected after endoscopic resection and included only patients with non-curative histopathological findings who subsequently underwent salvage surgery, whereas patients with curative endoscopic resection were not included. This introduces substantial selection bias and means that the study population was inherently enriched for adverse histopathological features. Consequently, the present analysis cannot be used to estimate the diagnostic performance of Paris morphology or pit-pattern classification in the broader population of patients undergoing pre-resection assessment for ESD/EID. Second, only five patients had SM1 invasion compared with 38 patients with SM2–3 invasion. This marked imbalance resulted in limited statistical power and considerable imprecision of the regression estimates. Third, the small sample size precluded robust multivariable modelling and formal assessment of diagnostic accuracy. Fourth, optical diagnosis is dependent on operator experience, imaging technology, and lesion characteristics. All procedures in the present study were performed at a single expert center, and the findings may therefore not be generalizable to other clinical settings. Accordingly, the results should be regarded as exploratory and hypothesis-generating rather than confirmatory.

In conclusion, in this selected cohort of patients with T1 colorectal cancer who underwent surgical treatment after non-curative ESD/EID, no statistically significant differences in Paris morphology or pit-pattern classification were demonstrated between lesions with SM1 and SM2–3 invasion. The marked imbalance between groups, the small number of SM1 cases, and the post-resection selection of the study population substantially limit inference. Accordingly, these findings should not be interpreted as estimates of the diagnostic accuracy of current endoscopic classification systems in an unselected population undergoing pre-resection endoscopic assessment. Rather, they identify residual uncertainty in the endoscopic characterization of invasion depth within a selected subgroup of non-curatively resected lesions and should be considered hypothesis-generating. Nevertheless, standardized pre-resection endoscopic characterization may retain clinical value by supporting patient counseling, multidisciplinary planning, and the development of integrated risk models, even when definitive indications for salvage surgery are established histopathologically. Prospective studies including consecutive patients with T1 colorectal cancer are required to determine whether complementary imaging techniques, artificial intelligence-assisted assessment, or biomarker-based approaches can further improve pre-resection risk stratification.

## Figures and Tables

**Table 1:** Baseline clinical and endoscopic characteristics according to depth of submucosal invasion (final histopathology).

Variable	SM1 (n = 5)	SM2–3 (n = 38)	*p* Value
Age (years), median (IQR)	60 (50–65)	65 (61–67)	0.262
Male sex, n (%)	4 (80.0%)	27 (71.1%)	1.000
BMI (kg/m^2^), median (IQR)	25.39 (21.80–26.54)	25.85 (23.73–29.14)	0.776
Rectal location, n (%)	2 (40.0%)	21 (55.3%)	0.650
Tumor size (mm), median (IQR)	40 (25–45)	30 (20.5–48.8)	0.690

Data are presented as median (IQR) or n (%). Percentages are calculated within each invasion-depth group. BMI body mass index; SM submucosal.

**Table 2:** Exploratory univariable associations between pre-resection characteristics and SM2–3 invasion.

Predictor	OR	95% CI	*p* Value
Tumor size (per 10 mm)	1.06	0.75–1.78	0.773
Rectal location	1.85	0.28–15.31	0.525
Paris flat morphology	2.57	0.38–21.38	0.332
Paris depressed morphology	0.22	0.02–5.39	0.259
Pit pattern V	2.33	0.27–16.62	0.395

OR odds ratio; CI confidence interval.

## Data Availability

The raw data supporting the conclusions of this article will be made available by the authors on request.

## Abbreviations

The following abbreviations are used in this manuscript:

ESD	Endoscopic Submucosal Dissection
EMR	Endoscopic Mucosal Resection
CRC	Colorectal Cancer
SM	Submucosal Invasion
EID	Endoscopic Intermuscular Dissection
